# Efficacy, safety, Low density lipoprotein cholesterol lowering, and calculated 10-year cardiovascular risk reduction of alirocumab and evolocumab in addition to maximal tolerated cholesterol lowering therapy: a post-commercialization study

**DOI:** 10.1186/s12944-017-0416-7

**Published:** 2017-01-23

**Authors:** Parth Shah, Charles J. Glueck, Naila Goldenberg, Sarah Min, Chris Mahida, Ilana Schlam, Matan Rothschild, Ali Huda, Ping Wang

**Affiliations:** 1 Graduate Medical Education Department, Jewish Hospital of Cincinnati, Cincinnati, Ohio USA; 2 The Jewish Hospital Internal Medicine Residency Program, 4777 E Galbraith Rd, Cincinnati, Ohio 45236 USA

**Keywords:** PCSK9 Inhibitor, Efficacy, Safety, Cardiovascular risk, Alirocumab, Evolocumab, Hypercholesterolemia, Low-density lipoprotein

## Abstract

**Background:**

Efficacy and safety of proprotein convertase subtilisin-kexin type 9 (PCSK9) inhibitors, alirocumab (ALI) and evolocumab (EVO) have previously been evaluated through controlled clinical trials with selective patient groups. Post-commercially, in patients with heterozygous familial hypercholesterolemia (HeFH) and/or cardiovascular disease (CVD) with suboptimal LDL cholesterol (LDLC) lowering on maximal tolerated cholesterol lowering therapy, we assessed efficacy and safety of ALI and EVO.

**Methods:**

Post-commercially, we started 25 patients on ALI 75 mg, 15 on ALI 150 mg, and 32 on EVO 140 mg bi-weekly added to entry LDLC lowering regimen, with follow-up for a median 24 weeks. History, physical exam, demographics, and adverse event data were collected. Changes in LDLC and AHA and NIH calculated 10-year CVD risks were assessed on ALI and EVO.

**Results:**

Of 72 patients, 25 had HeFH only, 25 CVD only, 22 had both, median age was 65 years, 63% females, 38% males, 86% Caucasian, 11% African-Americans, 17% diabetics, 63% on anti-hypertensives, and 7% smokers. At entry, 30 (42%) were on a statin and 42 (58%) could not tolerate any statins.

At 24-weeks, median LDLC decreased on ALI 75 mg from 117 to 62 mg/dL (−54%), on ALI 150 mg from 175 to 57 mg/dL (−63%), and on EVO 140 mg from 165 to 69 mg/dL (−63%), *p* <0.0001 for all. Absolute and percent LDLC reduction did not differ (*p* >.05) between ALI 150 and EVO 140 mg, but were less on ALI 75 mg vs ALI 150 mg and EVO 140 mg (*p* <.05).

Percent reductions in 10-year CVD risks by AHA and NIH calculators, respectively were ALI 75 mg −22 and −44%, ALI 150 mg −31 and −50%, and EVO 140 mg −29 and −56%, *p* ≤.002 for all.

The three most common adverse events included flu-like myositis 10%, respiratory tract symptoms 8%, and injection site reaction 6%.

**Conclusion:**

In patients with HeFH and/or CVD, LDLC was lowered by 63% on EVO and ALI 150 mg, and 54% on ALI 75 mg. Adverse events were minimal and tolerable. ALI and EVO represent paradigm shifts in LDLC lowering. Long term, post-commercial safety and efficacy remain to be determined.

## Background

With coronary artery disease and stroke being the number one cause of mortality in the United States [1], there is undeniable evidence that high LDL cholesterol (LDLC) contributes to an increased risk of cardiovascular disease (CVD). According to the CDC, approximately 78 million Americans (>21 years age) are eligible for cholesterol lowering medication, but only 55% are taking such therapy, of whom about 90% are on a statin [2]. Proprotein convertase subtilisin/kexin type 9 (PCSK9) inhibitors such as alirocumab (ALI) and evolocumab (EVO) have transformed LDLC lowering [3]. PCKS9 inhibitors are indicated in patients with heterozygous familial hypercholesterolemia (HeFH), Simon Broom’s Criteria [4] and/or WHO Dutch Lipid Criteria [5], and/or in patients with cardiovascular disease (CVD) whose LDLC lowering is suboptimal despite maximal tolerated cholesterol lowering therapy.

Previously, we have projected that an estimated 24 million Americans could be eligible for PCSK9 inhibitor therapy [6, 7]. Prior to commercialization, efficacy and safety of ALI and EVO in patients has been evaluated through randomized controlled clinical trials [8–12], which have stringent inclusion and exclusion criteria, creating a highly selective cohorts of study patients.

ALI ODYSSEY Phase III studies demonstrated that the mean percentage change in calculated low-density lipoprotein cholesterol (LDLC) from baseline to week 24 beyond statin effect was −61% versus 0.8% (placebo), *p* <0.001 [9, 13]. In 2,461 patients treated with ALI 150 mg, 796 (32%) had two consecutive LDLC levels <25 mg/dl while 288 (12%) had two consecutive LDLC levels <15 mg/dl [10]. In ODDYSSEY COMBO I, in addition to concurrent LDLC lowering therapy, ALI 75 mg produced a 45.6% LDLC decrease from baseline at 24 weeks [3]. Furthermore, in the OSLER-1 and OSLER-2 phase III trials, EVO 140 mg reduced LDLC by −61% at the median 12-week treatment mark, beyond statin effect. In a pool of 2,651 patients receiving EVO 140 mg, 1609 (61%) had at least one LDLC <25 mg/dl [11].

Compared to placebo in double-blind studies, there were minimal adverse reactions to the PCSK9 inhibitors with the difference being consistently <2% [9, 11].

Preliminary results of safety-efficacy controlled clinical trials, although not powered or designed to definitively assess CVD events, have revealed approximately 50% CVD risk reduction [9, 11].

Statin intolerance, predominantly characterized by myalgia, myositis, and myopathy, occurs in 10-29% of statin-treated patients [14, 15]. PCSK9 inhibitor therapy could substantially benefit these patients [6]. In the GAUSS-3 study of patients with previous statin intolerance, 43% of patients on atorvastatin had muscular symptoms. When these patients were enrolled in Phase B, which compared ezetimibe and placebo versus EVO and placebo, 29% experienced myalgias on ezetimibe versus 21% of those on EVO [8]. Furthermore, LDLC reduction from baseline on ezetimibe was −17% versus −53% on EVO at 24 weeks. In patients with statin intolerance, EVO was effective and well-tolerated [8].

Before 2013 [16], an LDLC target of <70 mg/dl was considered to be optimal for patients at high risk of coronary heart disease, and/or for type II diabetics. In treated patients from NHANES data, only 28% achieved LDLC < 70 mg/dl [17]. In 2014, the ACC/AHA guidelines [18] were no longer focused on LDLC targets <70 mg/dl, but suggested that high dose statins be given to patients matched to their calculated 10-year CVD event risk. It was suggested that LDLC be reduced by at least 50% in high risk patients, and by 30–49% in primary prevention of high risk patients [18]. In 2016, ACC Expert Consensus Decision Pathway re-introduced treatment thresholds, including LDLC <70 mg/dl in high risk patients with CVD [19]. For patients with clinically stable atherosclerotic cardiovascular disease (ASCVD), LDLC was targeted to <100 mg/dl and for those with CVD and comorbidities, LDLC targeted to <70 mg/dl [19].

Our specific aim, in a post-commercialization, open label, real world environment, was to assess the safety and efficacy of ALI and EVO in lowering LDLC, and subsequent change in calculated 10-year CVD risk in patients with HeFH and/or CVD referred to a regional cholesterol center for diagnosis and treatment of hypercholesterolemia.

## Methods

The procedures were in accordance with the ethical standards of human experimentation, and approved by The Jewish Hospital Institutional Review Board (JH#12-03, 15–06). Informed consent was provided and signed prior to initiation of the study.

Since the commercialization of PCSK9 inhibitors in July 2015, at our regional cholesterol center, 72 patients were started on either EVO or ALI based on qualifications for HeFH (HeFH, Simon Broom’s Criteria [4], WHO Dutch Lipid Criteria score >8 [5]) and/or CVD with suboptimal LDLC lowering despite maximal tolerated cholesterol lowering therapy, including statin doses down to zero. HeFH was assessed by the presence of tendon xanthomas and LDLC ≥190 mg/dl and/or personal or family history of premature cardiovascular disease and/or history of severe hypercholesterolemia. CVD was defined as carotid artery disease, history of stroke/TIA, coronary artery disease, congestive heart failure associated with CVD, and peripheral vascular disease.

Prior to initiation of therapy, all patients were counseled on a low cholesterol and saturated fat diet, and received follow-up counseling at serial visits. Instructions on how to use PCSK9 inhibitor auto-injector pens, education on its mechanism of action, side effects, and actions to be taken for missed doses were provided. Emergency contact information was given.

ALI and EVO were given in addition to patients’ entry maximal tolerated cholesterol lowering regimens. Insurance formulary coverage was taken into consideration when deciding whether to use ALI or EVO. If entry LDLC was ≤130 mg/dl, ALI 75 mg/ml was used, while for LDLC >130 mg/dl, ALI 150 mg was used, while EVO 140 mg was used for any entry LDLC. Sub-cutaneous auto-injector pens were used every two weeks.

All patients were followed for a median of 24 weeks, 25^th^-75^th^ percentile 24–28 weeks. We obtained a detailed history (especially cardiovascular history, documented HeFH and history of statin intolerance), physical examination, and lab draws at baseline and after starting therapy at 4, 12, and 24 weeks. Patient characteristics obtained included: age, gender, weight, body mass index, systolic and diastolic blood pressures, history of diabetes, smoking, and treatment with anti-hypertensive medications. Adverse events after the initiation of the therapy were recorded and appropriate changes were made as needed. The changes in 10-year cardiovascular risk were assessed using ACC/AHA [18] and NIH Framingham [20] risk calculators.

Statistical software SAS version 9.4 and Prism were used for data analysis and presentation. Paired Wilcoxon analysis was used to compare entry and follow-up data. General linear models were used to assess differences in LDLC lowering for the two ALI doses and EVO after adjusting for treatment duration, age, race, gender, BMI, presence or absence of statin intolerance, HeFH (+/−), and CVD (+/−). General linear models were also used to compare absolute and percent changes in LDLC in patients with and without entry statin intolerance, after adjusting for PCSK9 treatment type, treatment duration, age, race, gender, BMI, HeFH (+/−), CVD (+/−), and entry LDLC.

Absolute changes in LDLC were also assessed in a mixed effect model for repeated measures.

## Results

Table 1 displays entry characteristics of our cohort of 72 patients. Median age at entry was 65 years, 86% Caucasian, 11% African-American, 1% Asian, 1% Indian. Of the 72 patients, 63% were female, 38% male, 17% had diabetes, 7% smoked, and 63% were on anti-hypertensive medication. Of the 72 patients, 25 (35%) had HeFH only, 25 (35%) had CVD only, and 22 (31%) had both HeFH and CVD (Table 1). Of the 72 patients, 42 (58%) could not tolerate any dose of statin (Table 1). Before starting PCSK9 inhibitor therapy, 16 patients were taking a statin only, 5 statin and ezetimibe, 2 statin and colesevelam, 7 statin, ezetimibe, and colesevelam, and 11 ezetimibe and/or colesevelam (Table 1).

**Table 1:** 72 patients at study entry before treatment with Alirocumab or Evolocumab

Age at entry (years) Mean ± SD, [25^th^, 50^th^, 75^th^ percentiles]	64.1 ± 9.6, [58, 65, 72]
BMI (kg/m^2^) Mean ± SD, [25^th^, 50^th^, 75^th^ percentiles]	29.3 ± 5.1, [25.3, 29.0, 32.2]
Race	62 White (86%), 8 Black (11%), 1 Asian (1%), 1 Indian (1%)
Gender	45 F (63%), 27 M (38%)
Diabetes	12 Yes (17%), 60 No (83%)
Smoke	5 Yes (7%), 67 No (93%)
BP lowering drug	45 Yes (63%), 27 No (38%)
HeFH	47 Yes (65%), 25 No (35%); 25 had HeFH & no CVD (35%)
CVD	47 Yes (65%), 25 No (35%); 25 had CVD & no HeFH (35%)
Both HeFH & CVD	22 (31%)
Statin intolerant	42 Yes (58%), 30 No (42%)
Medication use at entry	Statin only, *N* = 16
Taking Statin (n = 30)	Statin + ezetimibe, *N* = 5
	Statin + colesevelam, *N* = 2
	Statin + ezetimibe + colesevelam, *N* = 7
Not taking statin (*n* = 42)	Ezetimibe only, *N* = 4
	Colesevelam only, *N* = 2
	Ezetimibe + colesevelam, *N* = 5
	Nothing, *N* = 31
Follow up weeks on ALI or EVO Mean ± SD, [25^th^, 50^th^, 75^th^ percentiles]	26 ± 5, [24, 28]

**Fig. 1:**
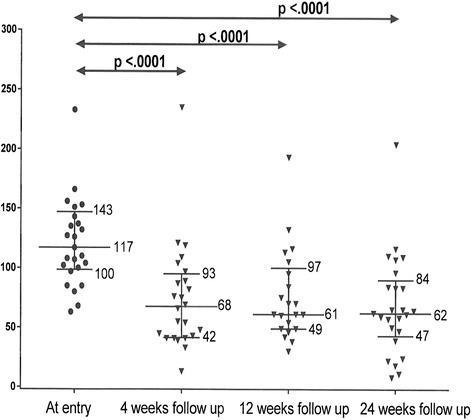
Median and 25^th^ and 75^th^ percentile LDLC (mg/dl) at study entry and 4, 12, and 24 weeks follow-up on Alirocumab 75 mg every two weeks

Tables 2, 3, and 4 display 25^th^, 50^th^, and 75^th^ percentiles for LDLC and total cholesterol, triglyceride, and HDL cholesterol categorized by drug group and further characterized by HeFH and CVD, Table 3. On ALI 75 mg, entry LDLC fell from a median of 117–62 mg/dl, a 54% decrement, Table 2. On ALI 150 mg, entry LDLC fell from a median of 175–57 mg/dl, a 63% reduction, and on EVO 140 mg, entry LDLC fell from 165 to 69 mg/dl, a 63% reduction, Table 2, Fig. 1. Median, 25^th^ and 75^th^ percentiles for follow-up were 24, 24, and 28 weeks, Table 1.

**Table 2:** Changes in LDLC and CVD risk from study entry to last follow up in 72 patients taking Alirocumab or Evolocumab

		Alirocumab 75 mg (*n* = 25) Follow up length median 24 weeks			Alirocumab 150 mg (*n* = 15) Follow up length median 26 weeks			Evolocumab 140 mg (*n* = 32) Follow up length median 24 weeks		
Variable measured		percentile			percentile			percentile		
		25^th^	50^th^	75^th^	25^th^	50^th^	75^th^	25^th^	50^th^	75^th^
LDLC	Entry (mg/dl)	100	117	143	133	175	214	143	165	211
	Follow up (mg/dl)	47	62	84	49	57	86	46	69	109
	Absolute change (mg/dl)	−35	−67	−85	−89	−104	−141	−65	−89	−131
	P (paired Wilcoxon)	*p* <.0001			*p* <.0001			*p* <.0001		
	Percent change (%)	−27	−54	−63	−56	−63	−72	−40	−63	−71
	P (Wilcoxon)	*p* <.0001			*p* <.0001			*p* <.0001		
CVD risk for next 10 years With AHA calculator	Entry (%)	3.9	6.2	18.0	5.4	9.3	20.4	4.3	11.5	18.6
	Follow up (%)	3.3	6.2	10.1	2.3	7.0	15.1	2.9	6.7	20.2
	Absolute change	−0.2	−1.6	−5.4	−0.7	−3.3	−6.0	−0.6	−2.4	−5.7
	P (paired Wilcoxon)	*p* = .0001			*p* = .0043			*p* = <.0001		
	Percent change	−1.9	−22.2	−40.7	−22.2	−31.3	−39.0	−9.1	−28.7	−52.3
	P (Wilcoxon)	*p* = .0002			*p* = .0015			*p* <.0001		
CVD risk for next 10 years With NIH calculator	Entry (%)	6.8	11.2	19.8	10.6	17.2	25.7	9.6	17.4	26.4
	Follow up (%)	4.3	7.4	11.7	5.1	6.5	12.3	5.3	8.0	12.0
	Absolute change	−1.4	−4.2	−10.5	−4.4	−9.1	−16.2	−3.0	−7.1	−14.7
	P (paired Wilcoxon)	*p* <.0001			*p* = .0001			*P* <.0001		
	Percent change	−21.4	−43.7	−53.5	−41.6	−49.8	−61.4	−27.9	−55.5	−66.2
	P (Wilcoxon)	*p* <.0001			*p* = .0001			*p* <.0001

**Table 3:** Number (%) of patients who had at least one measure of LDLC <70 mg/dl on Alirocumab or Evolocumab for 24 weeks

	HeFH only (*n* = 25) Entry LDLC 25^th^, 50^th^, 75^th^%tile: [149, 177, 220 mg/dl]	CVD only (*n* = 25) [104, 131, 148 mg/dl]	HeFH & CVD (*n* = 22) [122, 169, 214 mg/dl]	Total cohort (*n* = 72) [123, 149, 193 mg/dl]
Alirocumab 75 mg/2 weeks (*n* = 25)	2/5 (40%)	12/14 (86%)	5/6 (83%)	19/25 (76%)
Alirocumab 150 mg/2 weeks (*n* = 15)	2/4 (50%)	3/3 (100%)	6/8 (75%)	11/15 (73%)
Evolocumab 140 mg/2 weeks (*n* = 32)	8/16 (50%)	7/8 (88%)	3/8 (38%)	18/32 (56%)
All 3 treatment groups (*n* = 72)	12/25 (48%)	22/25 (88%)	14/22 (64%)	48/72 (67%)

**Table 4:** Change in total cholesterol, triglyceride, and HDL cholesterol in 72 patients treated with Alirocumab or Evolocumab

		Alirocumab 75 mg (*n* = 25) Follow up length median 24 weeks			Alirocumab 150 mg (*n* = 15) Follow up length median 26 weeks			Evolocumab 140 mg (*n *= 32) Follow up length median 24 weeks		
Variable measured		percentile			percentile			percentile		
		25^th^	50^th^	75^th^	25^th^	50^th^	75^th^	25^th^	50^th^	75^th^
Total cholesterol	Entry (mg/dl)	172	192	231	227	259	294	222	252	299
	Follow up (mg/dl)	118	155	177	114	145	181	117	157	203
	Absolute change (mg/dl)	−25	−76	−94	−83	−105	−168	−65	−86	−139
	P (paired Wilcoxon)	*p* <.0001			*p* <.0001			*p* <.0001		
	Percent change (%)	−14	−32	−45	−34	−48	−53	−26	−39	−52
	P (Wilcoxon)	*p* <.0001			*p* <.0001			*p* <.0001		
Triglyceride	Entry (mg/dl)	96	135	173	124	160	317	101	145	167
	Follow up (mg/dl)	80	106	154	76	105	161	80	106	142
	Absolute change (mg/dl)	+3	−29	−57	−10	−51	−102	+4	−25	−52
	P (paired Wilcoxon)	*p* = .0051			*p* = .0015			*p* = .0069		
	Percent change (%)	+3	−21	−33	−12	−32	−41	+4	−23	−35
	P (Wilcoxon)	*p* = .0097			*p* = .0015			*p* = .0092		
HDL cholesterol	Entry (mg/dl)	41	53	61	40	51	57	45	56	68
	Follow up (mg/dl)	40	51	65	44	52	65	47	58	75
	Absolute change (mg/dl)	−1	+2	+5	+1	+7	+10	0	+4	+14
	P (paired Wilcoxon)	*p* = .070			*p* = .0075			*p* = .0028		
	Percent change (%)	−2	+5	+11	+3	+11	+17	0	+8	+21
	P (Wilcoxon)	*p* = .092			*p* = .010			*p* = .0029

**Fig. 2:**
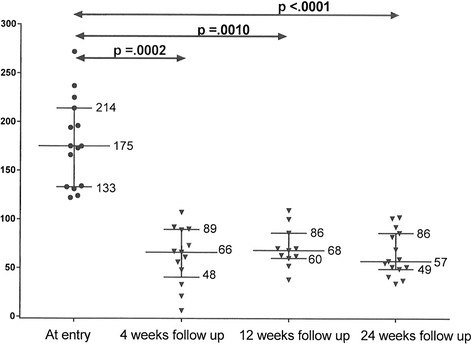
Median and 25^th^ and 75^th^ percentile LDLC (mg/dl) at study entry and 4, 12, and 24 weeks follow-up on Alirocumab 150 mg every two weeks

**Fig. 3:**
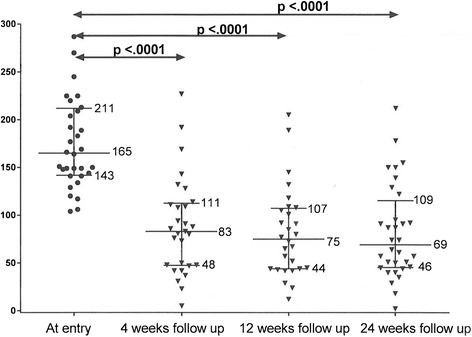
Median and 25^th^ and 75^th^ percentile LDLC (mg/dl) at study entry and 4, 12, and 24 weeks follow-up on Evolocumab 140 mg every two weeks

Figures 1, 2, 3 display 25^th^, 50^th^, and 75^th^ percentiles of LDLC levels at study entry and 4, 12, and 24 weeks follow-up in the ALI 75 mg, ALI 150 mg, and EVO 140 mg groups. Median LDLC in the ALI 75 mg group decreased from 117 to 68 mg/dl at 4 weeks, 61 mg/dl at 12 weeks, and 62 mg/dl at 24 weeks (*p* <.0001 for all). Median LDLC in the ALI 150 mg group decreased from 175 to 66 mg/dl at 4 weeks, 68 mg/dl at 12 weeks and 57 mg/dl at 24 weeks (*p* <.0001 overall, Fig. 2). Median LDLC in the EVO 140 mg group decreased from 165 to 83 mg/dl at 4 weeks, 75 mg/dl at 12 weeks, and 69 mg/dl at 24 weeks (*p* <.0001 for all, Fig. 3). For each drug group, there was a sharp reduction in LDLC by 4 weeks, which remained stable at 12 and 24 weeks (Figs. 1, 2, 3).

In the mixed effect model for repeated measures, LDLC reductions on ALI 75, ALI 150, and EVO 140 mg were significant, *p*<. 0001 for all.

Table 3 demonstrates the number of patients who had attained at least one measurement of LDLC <70 mg/dl while on ALI 75 mg, ALI 150 mg, or EVO 140 mg. For the total cohort, median entry LDLC was 149 mg/dl, 177 mg/dl for HeFH only, 131 mg/dl for CVD only, and 169 mg/dl for both HeFH and CVD, Table 3. Of the 25 patients in the HeFH only group, 12 (48%) achieved LDLC <70 mg/dl, Table 3. Of the 25 patients with CVD only, 22 (88%) achieved LDLC <70 mg/dl. Of the 22 patients with both HeFH and CVD, 14 (64%) achieved LDLC <70 mg/dl, Table 3.

As displayed in Table 3, of the 25 patients on ALI 75 mg, 19 (76%) achieved LDLC <70 mg/dl. Of the 15 patients on ALI 150 mg, 11 (73%) achieved LDLC <70 mg/dl. Of 32 patients on EVO 140 mg, 18 (56%) achieved LDLC <70 mg/dL. In the total cohort of 72 patients, 48 (67%) achieved LDLC <70 mg/dl, Table 3.

As shown in Table 4, reductions in LDLC were accompanied by median reduction in total cholesterol of 32% (ALI 75 mg), 48% (ALI 150 mg), and 39% (EVO 140 mg), *p* < .0001 for all. On ALI 75 mg, ALI 150 mg, and EVO 140 mg, there were also significant median reductions in triglycerides (TG) of 21%, 32%, and 23% (*p* = .01, *p* = .002, *p* = .009), respectively (Table 4). Median HDLC increased 5% on ALI 75 mg, 11% on ALI 150 mg and 8% on EVO 140 mg (*p* = .092, *p* = .010, *p* = .003, Table 4).

By stepwise regression, among age, race, BMI, gender, statin intolerance (+/−), HeFH (+/−), CVD (+/−), PCSK9 treatment type, and follow-up duration, the only significant explanatory variable for absolute change or percent change in LDLC was PCSK9 treatment type. Using a general linear model, adjusting for age, race, BMI, gender, statin intolerance (+/−), HeFH (+/−), CVD (+/−) and follow-up duration, absolute and percent LDLC reduction were greater with ALI 150 mg than with ALI 75 mg (*p* = .004, *p* = .011), and greater with EVO 140 mg than with ALI 75 mg (*p* = .014, *p* = .044), Table 5. ALI 150 mg and EVO 140 mg did not differ in regards to LDLC reduction (*p* >0.05). In a second general linear model, covariance adjusted absolute and % reduction in LDLC on therapy was greater in the 30 statin-tolerant patients who continued statins plus ALI or EVO for 24 weeks, versus the 42 patients with statin intolerance, *p* = .0008, *p* = .013, Table 5.

**Table 5:** Comparisons of LDLC change among PCSK9 inhibitor treatment groups and between statin tolerant and intolerant groups

	Alirocumab 75 mg (*n* = 25)	Alirocumab 150 mg (*n* = 15)	Evolucumab 140 mg (*n* = 32)
LS means ± SE of change in LDLC (mg/dl)	−59 ± 10	−110 ± 14	−95 ± 9
Group differences	ALI 75 vs ALI 150, p = .004		
	ALI 75 vs EVO 140, p = .014		
LS means ± SE of % change in LDLC (%)	−42 ± 5%	−63 ± 7%	−56 ± 4%
Group differences	ALI 75 vs ALI 150, p = .011		
	ALI 75 vs EVO 140, p = .044		
LS means ± SE of change in LDLC (mg/dl)	−109 ± 7	−78 ± 5	
Group differences	p = .0008		
LS means ± SE of % change in LDLC (%)	−63 ± 5%	−48 ± 4%	
Group differences	*p* = .013		

LS means for 3 treatment groups, adjusted for treatment duration, age, BMI, race, gender, statin intolerance (yes-no), HeFH (yes-no), and CVD (yes-no)

LS means for statin tolerant vs intolerant groups, adjusted for PCSK9 groups, treatment duration, age, BMI, race, gender, HeFH (yes-no), CVD (yes-no) and LDLC at entry

Reductions in LDLC were accompanied by significant absolute and percent reductions in estimated 10-year CVD risk as determined by both the ACC/AHA and the NIH calculators (Table 2). On ALI 75 mg, ALI 150 mg, and EVO 140 mg, by the NIH calculator, at last follow-up, 10-year CVD risk was reduced by median of 44, 50 and 56%, *p* ≤.0001 for all. By the AHA calculator, at last follow-up, 10-year CVD risk was reduced by median of 22, 31 and 29%, *p* <.002 for all (Table 2).

Table 6 displays adverse events (AE) in the 72 patients on the 3 treatment regimens. The three most common adverse events were a flu-like myositis (10%), respiratory tract symptoms/infection (8%), and injection site reaction (6%), Table 6. As displayed in Table 6, there were no significant differences among the 3 groups for AEs (*p* >0.05).

**Table 6:** Adverse events in 72 patients on Alirocumab 75 or 150 mg, or Evolocumab 140 mg, by treatment regimens

	All 3 treatment groups (*n *= 72) F45, M27 Follow up length median 25 weeks	Alirocumab 75 mg (*n* = 25) F12, M13 Follow up length median 24 weeks	Alirocumab 150 mg (*n* = 15) F10, M5 Follow up length median 26 weeks	Evolocumab 140 mg (*n* = 32) F23, M9 Follow up length median 24 weeks
Flu-like myositis	8 (10%)	1 (4%)	5 (33%)	2 (6%)
Respiratory tract infection/symptoms	6 (8%)	1 (4%)	1 (7%)	4 (13%)
Inject site reaction	4 (6%)	1 (4%)	1 (7%)	2 (6%)
Fatigue	1 (1%)	1 (4%)		
Headache/mental acuity/mood	2 (3%)		1 (7%)	1 (3%)
Urticaria/itchiness	2 (3%)	1 (4%)		1 (3%)
G.I. symptom	2 (3%)		1 (7%)	1 (3%)
Weight gain	1 (1%)			1 (3%)
Hair loss	1 (1%)			1 (3%)
Any adverse events	22 (31%)	5 (20%)	7 (47%)	10 (31%)
No adverse events	50 (69%)	20 (80%)	8 (53%)	22 (69%)

Comparing adverse events (any vs none), no difference among 3 treatment groups (Fisher’s* p* > .05)

As displayed in Table 7, the statin tolerant group, taking a statin plus ALI or EVO, had fewer AEs than the statin intolerant group, taking ALI or EVO only, *0* = .039.

**Table 7:** Adverse events in 72 patients on Alirocumab or Evolocumab, by entry statin intolerance group

	All (*n* = 72) F45, M27 Follow up length median 25 weeks	Statin tolerant, taking statin (*n* = 30) F15, M15 Follow up length median 24 weeks	Statin intolerant (*n* = 42) F30, M12 Follow up length median 23 weeks
Flu-like myositis	8 (10%)	1 (3%)	7 (17%)
Respiratory tract infection/symptoms	6 (8%)	2 (7%)	4 (9%)
Inject site reaction	4 (6%)	2 (7%)	2 (5%)
Fatigue	1 (1%)		1 (2%)
Headache/mental acuity/mood	2 (3%)		2 (5%)
Urticaria/itchiness	2 (3%)		2 (5%)
G.I. symptom	2 (3%)		2 (5%)
Weight gain	1 (1%)		1 (2%)
Hair loss	1 (1%)		1 (2%)
Any adverse events	22 (31%)	5 (17%)	17 (40%)
No adverse events	50 (69%)	25 (83%)	25 (60%)

Comparing adverse events (any vs none), there were fewer adverse events in the statin tolerant group, taking statin + ALI or EVO than in the statin intolerant group taking ALI or EVO only (Fisher’s *p* = .039)

One patient had coronary bypass revision due to scar tissue growth within one month of starting therapy and another patient had three stents placed within two months of starting therapy. In neither of the cardiovascular event patients was the PCSK9 inhibitor therapy stopped and we did not attribute these two events to the PCSK9 inhibitor therapy.

## Discussion

After taking into account the rising CVD costs in the United States, projected by the AHA to be approximately $1 trillion by 2030, we have postulated that the cost to society with an estimated 50% CVD risk reduction with PCSK9 inhibitor therapy [6, 10, 11] would be in the middle of the range of societal costs for CVD [6]. Subsequently, in 103 hypercholesterolemic patients [7] (61 with previous CVD events, first CVD event at median age 55, median LDLC 139 mg/dL despite maximal tolerated cholesterol-lowering therapy), we estimated direct and indirect costs of CVD, cost of estimated next 10-year CVD events, and PCSK9 inhibitor costs to assess whether PCSK9 inhibitors would provide an incremental cost-effectiveness ratio [21] within a society willingness to pay threshold [22]. We concluded [7] that the net cost of PCSK9 inhibitor therapy, assuming a 50% reduction of CVD events on PCSK9 inhibitor therapy, was $7,000 per patient per year in the past, and the net cost of therapy over the next 10 year period was estimated to be $12,459 per patient per year, well below the $50,000 per quality adjusted life year [22] gained which has been used to judge value of a pharmacologic therapy.

Despite maximal tolerated cholesterol lowering therapy, many patients fail to achieve optimal LDLC lowering [23–25], with only 28% of patients in NHANES achieving LDLC <70 mg/dl on treatment [17]. Failure to reach optimal LDLC lowering is related to statin intolerance [26, 27], expense, lack of insurance coverage, or variations in statin availability across states in insurance, race, and ethnicity [23]. In the current study, 42 of 72 patients (58%) were statin intolerant, a problem which affects at least 10-29% of patients taking statins [14, 15, 28]. Moreover 60% of patients who discontinue statins report statin intolerance as the reason [29]. However, congruent with our open label, post commercialization study, as demonstrated by the controlled clinical trial, GAUSS-3, in patients with statin intolerance, EVO was well-tolerated and effective [8]. PCSK9 inhibitors now offer the promise of optimizing LDLC in most patients with HeFH, CVD, and concurrent statin intolerance [9–12, 30–32].

ALI and EVO have been found to be very efficacious and safe during phase II and III randomized controlled trials with minimal adverse events compared to placebo [8, 9, 11]. In the phase II MENDEL study, without a concurrent lipid-lowering regimen, EVO 140 mg showed a 51% reduction in LDLC at 12 weeks [33]. In SAR236553/REGN727 phase II trials with ALI 150 mg added on a stable atorvastatin dose, there was 72% LDLC reduction at 12 weeks [34]. During phase III trials with patients on maximal tolerated cholesterol lowering therapy along with ALI 150 mg and ALI 75 mg every two weeks, there was a 61 and 46% reduction, respectively, from baseline in LDLC at median 24 weeks [3, 9]. In OSLER-1 and 2, patients on EVO 140 mg every two weeks or 420 mg once/month had LDLC reduction by 61% at median 12 weeks on top of antecedent cholesterol lowering therapy [11].

Based on FDA indications and third party insurance drug coverage, our current study was done in HeFH and CVD patients with suboptimal cholesterol lowering despite maximal tolerated cholesterol lowering therapy. This qualified all our cohort, with minimal exclusion criteria, for initiation of PCSK9 inhibitor therapy, a cohort much more diverse than those in the placebo-controlled randomized clinical trials [9, 11]. Rallidis et al. have recently demonstrated that in patients who presented with myocardial infarction, 20% had definite/probable HeFH and 51% had possible HeFH [35]. Over a 9-year follow-up period, 39% of 255 patients had a major adverse coronary event despite 84.3% being on statins, with only 2.3% achieving LDLC <70 mg/dl [35]. Definite/probable HeFH was independently associated with major adverse coronary events [35]. Our current study cohort included 25 patients with HeFH only, 25 with CVD only, and 22 with both. Moreover, in our current study, 48/72 (67%) patients obtained optimal LDLC reduction to <70 mg/dl while taking ALI or EVO. Of the 25 patients with HeFH only, 12 (48%) had ≥ 1 LDLC < 70 mg/dl on therapy, as did 22 of 25 (88%) patients with CVD only, and 14 of 22 (64%) with both HeFH and CVD. Our findings support the central importance of PCSK9 inhibitor therapy in high-risk patients with HeFH and/or CVD who otherwise do not achieve LDLC <70 mg/dl with maximal tolerated LDLC lowering regimens.

In our current study, median absolute reductions in LDLC on ALI 75 mg, ALI 150 mg, and EVO 140 mg were respectively 67, 104, and 89 mg/dl, which, if maintained, should lead to sharp reductions in CVD events. In our current study, median LDLC reduction from baseline was 54% and 63% on ALI 75 and 150 mg, and 63% on EVO 140 mg respectively at 24 weeks. Collins et al. reported that reduction of LDLC by 77 mg/dl for 5 years in 10,000 patients would prevent major vascular events in 1,000 (10%), an absolute benefit in those who had pre-existing CVD, and in 500 patients (5% absolute benefit) in primary prevention [36]. In the 30 statin-tolerant patients in our current study, subsequently receiving both statins and ALI or EVO, absolute and percent LDLC reduction was greater than in the 42 patients with statin intolerance at entry who subsequently received only ALI or EVO. LDLC reduction in our real world setting for ALI 150 mg and EVO 140 mg was within 1-2% of that reported in the placebo controlled trials while in the ALI 75 mg group it was about 9% higher than the previous trials [9, 11].

From past vascular studies on statins, regression of plaque can be induced when LDLC is held ~70 mg/dl or below [37]. Patients receiving atorvastatin 80 mg have been shown to have regression of carotid-artery intima-media within 12 months with an average achieved LDLC of 76 mg/dl [38]. The NIH post-CABG study showed that patients who were post CABG and selected for a lower target LDLC group (90 mg/dl vs 135 mg/dl) had no angiographic progression of coronary plaques. This suggested that lower target LDLC leads to a significant reduction in coronary events and mortality, inferring that further reduction of coronary plaque burden could lead to further reduced coronary event rates and mortality [39]. When patients were given rosuvastatin 40 mg in the ASTEROID trial, mean LDLC was reduced from 130 mg/dl to 60 mg/dl (53%) with a total atheroma volume reduction median of 6.8% as well as a significant reduction in all intravenous ultrasound measurements of atheroma burden [40]. Consequently, in the recent GLAGOV study [41], compared with statin-placebo, the EVO-statin group achieved lower mean LDLC (93.0 vs 36.6 mg/dL, *p* <.001). The primary efficacy parameter, percent atheroma volume (PAV), increased 0.05% with placebo and decreased 0.95% with EVO, *p* <.001). EVO induced plaque regression in more patients than placebo (64.3% vs 47.3%, *p* <.001 for PAV, and 61.5% vs 48.9%, *p* <.001 for total atheroma volume (TAV)). The GLAGOV study also demonstrated a positive linear change in percent PAV as LDLC increased from 20 mg/dl to 110 mg/dl.

Mendelian randomization studies suggest that a lifetime reduction of LDLC ~40 mg/dl would reduce risk of ASCVD by 50% [42]. In our current study, the median LDLC reduction ranged from 67 to 104 mg/dl, and 48 (67%) of 72 patients achieved at least one LDLC on therapy <70 mg/dl. Moreover, according to the AHA and NIH 10-year CVD risk calculations, on ALI 75 mg there was CVD risk reduction of 22 and 44%, on ALI 150 mg, 31 and 50%, and on EVO 140 mg, 29 and 56%. The ACC/AHA calculator was not, however, designed for use in patients with pre-existing CVD events, although the NIH calculator has no such restriction [43]. In at least 50% of our patients on PCSK9 therapy with LDLC < 70 mg/dl, from the past experience with vascular studies on statins [37, 38, 40] and recent GLAGOV study [38], we speculate that there should be significant regression of vascular plaque. Although not powered for CVD outcomes, the preliminary randomized controlled trials outcomes’ data for ALI and EVO showed 50% CVD event reduction [9–11]. Further hard CVD endpoint as well as vascular regression studies [41] are needed to assess for cardiovascular impact of the powerful LDLC reduction from PCSK9 therapy.

Assessment of potential adverse events is an important consideration when analyzing PCSK9 inhibitor use. In the current study, both ALI and EVO were generally well-tolerated; the most significant frequent adverse event was flu-like myositis-myalgia in 10% of patients. There were however, no among-group differences between all three treatment groups for adverse events (*p* >0.05). This is comparable to the pattern of side effects for ALI and EVO in randomized placebo-controlled trials [44, 45]. In a meta-analysis of 25 randomized controlled trials with PCSK9 inhibitors, there was no significant differences in major adverse event rates between the active drug and control treatment [46].

In the current study, of the 42 statin-intolerant patients, defined primarily as experiencing myalgias or myopathy on statins, 17% had mild to moderate flu-like myalgias. When comparing the statin-intolerant cohort on ALI or EVO versus those taking a statin plus ALI or EVO, there were fewer adverse events in the statin tolerant group than the statin intolerant group (*p* = .04). Our results parallel those of the randomized, placebo-controlled GAUSS-3 study, where EVO was well-tolerated and effective in patients with statin intolerance [8]. Our conjecture is that the long-term adverse health consequences attributed to PCSK9 inhibitors may be minimal, particularly in statin tolerant patients.

In our real world, open label, post-commercialization evaluation of ALI and EVO, with 58% of patients being statin intolerant, and all having HeFH and/or CVD, our LDLC reduction was within 1-3% of the placebo-controlled trials for ALI 150 mg and EVO 140 mg groups, but the ALI 75 mg group had 9% higher LDLC lowering than earlier trials [9, 11]. A second strength of our study was the finding that the adverse event profile on ALI or EVO was slightly lower in the statin tolerant patients taking both statins and ALI or EVO versus the statin intolerant patients taking only ALI or EVO. Since 58% of the cohort was intolerant to any statin at any dose regimen at entry, this emphasizes both the high frequency of statin intolerance in patients with high LDLC who fail to reach LDLC goals, and the efficacy of ALI and EVO inhibitors in stain intolerant patients. A third strength lies in the characterization of those patients who achieved LDLC <70 mg/dl by each treatment group, and in the presence or absence of HeFH and/or CVD.

A limitation of this study is the relatively small group of patients. A second limitation is a probable bias towards higher risk patients with HeFH, CVD, and statin intolerance, unable to reach LDLC lowering goals on conventional LDLC-lowering therapy, by virtue of referral to a regional cholesterol treatment center.

## Conclusion

In hypercholesterolemic patients with HeFH, and/or CVD with suboptimal LDLC lowering on maximal tolerated cholesterol lowering therapy, LDLC was reduced by 63% on EVO 140 mg and ALI 150 mg and 54% on ALI 75 mg beyond the best antecedent cholesterol lowering program. Reported adverse events were minimal and tolerable. ALI and EVO represent paradigm shifts in LDLC lowering, and, speculatively, in reduction of CVD, with long-term safety and cardiovascular outcomes yet to be determined.

## Abbreviations

ACC: American college of cardiology
AHA: American heart association
ALI: Alirocumab
ASCVD: Atherosclerotic cardiovascular disease
CVD: Cardiovascular disease
EVO: Evolocumab
HeFH: Heterozygous familial hypercholesterolemia
IVUS: Intravenous ultrasound
LDLC: Low-density lipoprotein cholesterol
NIH: National institute of health
PCSK9: Proprotein convertase subtilisin-kexin type 9
TIA: Transient ischemic attacks
WHO: World health organization
